# Inputs and outputs of poly(ADP-ribosyl)ation: Relevance to oxidative stress

**DOI:** 10.1016/j.redox.2014.08.003

**Published:** 2014-08-21

**Authors:** Csaba Hegedűs, László Virág

**Affiliations:** a Department of Medical Chemistry, Faculty of Medicine, University of Debrecen, Nagyerdei Krt. 98., H-4032 Debrecen, Hungary; b MTA-DE Cell Biology and Signaling Research Group, Debrecen, Hungary

**Keywords:** Poly(ADP-ribosyl)ation, PARP1, Oxidative stress, Cell death, Necroptosis, Apoptosis

## Abstract

Oxidative stress can cause DNA breaks which induce activation of the DNA nick sensor enzyme poly(ADP-ribose) polymerase-1 (PARP-1), part of the 17 member PARP enzyme family. PARP-1 modifies target proteins by attaching to them several NAD-derived ADP-ribose units forming poly(ADP-ribose) (PAR) polymers. PARylation controls many cellular processes while intense PARylation may also lead to cell death by various mechanisms. Here we summarize the modes of activation, inhibitors and modulators of PARP-1 and review the cellular functions regulated by the enzyme.

## Introduction

Poly(ADP-ribosyl)ation (PARylation) is a protein modification catalyzed by poly(ADP-ribose) polymerase (PARP) enzymes. The enzyme family was created based on database screening using the highly conserved PARP signature motif. Whereas the PARP enzyme family consists of 17 members [1], only three of these enzymes can be considered as bona fide PARPs while the other family members function as mono(ADP-ribosyl) transferases or their enzymatic activity has not yet been characterized. PARylation requires NAD as substrate and this energy metabolite is cleaved into nicotinamide and ADP-ribose by active PARP enzymes. In turn, PARPs attach the first ADP-ribose unit to appropriate substrates and then generate further ADP-ribose units by repeated NAD cleavage and polymerize ADP-ribose moieties [2].

Here we review the mechanisms by which activity of PARP-1 can be stimulated, inhibited or modulated. We also aim to summarize the cellular functions that are regulated by PARP-1.

## Routes for PARP-1 activation

**Fig. 1:**
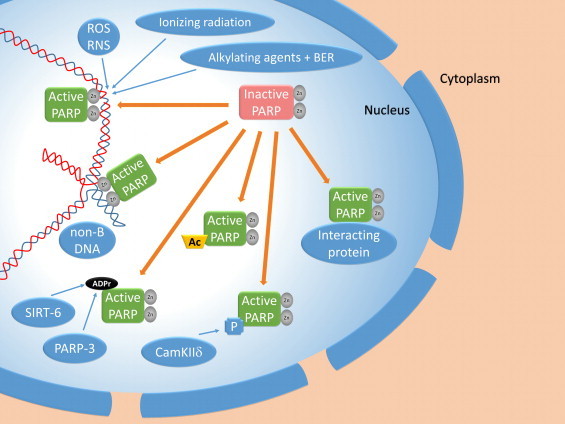
Activation of PARP-1. The nuclear enzyme PARP-1 can bind to DNA breaks resulting in the activation of the enzyme. DNA breaks are caused either by direct attacks by ROS, RNS or ionizing radiation or may form indirectly when the DNA repair machinery introduces breaks into the DNA strands following e.g. alkylating DNA damage. Binding to non-B DNA structures such as bent or cruciform DNA or four-way junctions may also lead to PARP-1 activation. DNA independent activation mechanisms have also been described for PARP-1. These include protein–protein interactions or covalent modifications (e.g. mono-ADP-ribosylation, acetylation or phosphorylation) (for details and references see text).

PARP-1 has originally been described as a DNA nick sensor enzyme activated by DNA single and double strand breaks [3]. DNA damage-induced PARP-1 activation is considered as the classical route for the activation of the enzyme (Fig. 1). PARP-1 binds to broken DNA ends via the zinc finger motives found in the N-terminal DNA binding domain. Reactive oxygen and nitrogen species (ROS and RNS, respectively) activate PARP-1 via this route as many ROS/RNS species are capable of causing DNA single strand breaks [4]. Ionizing radiations may also cause DNA breaks either directly (e.g. alpha particles or neutrons which have high linear energy transfer) or indirectly (via interaction with water resulting in the production of hydroxyl radicals). Moreover, repair of damage caused by alkylating agents [e.g. N-methyl-N-nitro-N-nitrosoguanidine (MNNG), N-nitroso-N-methylurea (MNU), temozolomide, and carmustine] also feed into this route as DNA repair machineries (e.g. base excision repair and nucleotide excision repair) introduce cuts (single or double strand breaks) leading to PARP-1 activation [5].

The findings that stimuli other than broken DNA can also activate PARP-1 (Fig. 1) led to a paradigm shift in the investigation of the enzyme [6]. Lonskaya et al. showed that PARP-1 can bind to non-B DNA structures (three- and fourway junctions, hairpins, cruciforms and stably unpaired regions) resulting in activation of the enzyme [7]. Moreover, the enzyme may be activated by interactions with partner proteins (Fig. 1). For example interaction with the N-terminal tail of histone 4 has been shown to activate PARP-1 [8]. Moreover, physical interaction between PARP-1 and the phosphorylated form of Erk MAP kinase also activates PARP-1 [9,10]. Furthermore, protein modifications, e.g. phosphorylation by certain protein kinases such as CamKII delta [11], mono-ADP-ribosylation by SIRT6 [12,13] or PARP-3 [14] or acetylation can also stimulate PARP-1 activity [15] (Fig. 1). Of note, a basal phosphorylation by an unknown kinase was found to be required for PARP-1 activity [16]. SIRT6 a mammalian homolog of the yeast Sir2 deacetylase has been shown to be recruited to the sites of oxidative DNA damage (double strand breaks) where it associates with PARP1 and activates it by mono-ADP-ribosylation [13]. PARP-3 can also catalyze activating mono(ADP-ribosyl)ation of PARP-1 but this reaction takes place in the absence of DNA [14]. PARP-1 has also been shown to be a target of acetylation [17]. Acetylation of PARP-1 may contribute to the maintenance of the active state of the enzyme as deacetylation by SIRT-1 downregulated the activity of PARP-1 [15].

## Inhibitors and modulators of PARP-1

**Fig. 2:**
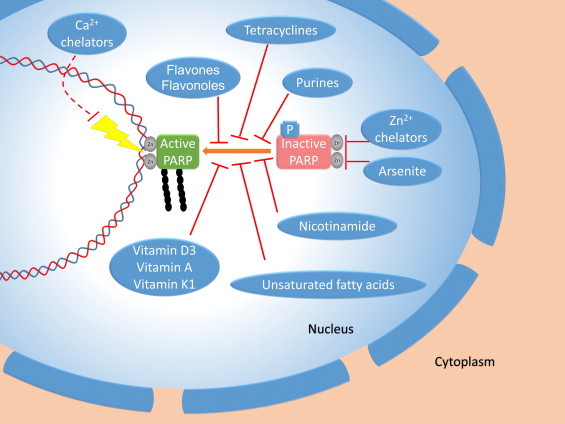
Inhibition of PARP-1. PARP-1 is an attractive pharmacological target in various diseases and therefore several classes of small molecule PARP inhibitors (not shown) have been developed and tested successfully in cellular assays and animal models of diseases ranging from cancer, ischemia-reperfusion injury, stroke, sepsis to shock. Several biomolecules such as flavones, flavonols, vitamins D3, A and K, purines and even unsaturated fatty acids have been described to possess PARP inhibitory activities. Other bioactive compounds such as arsenite or tetracyclines also inhibit PARP-1. Inhibiting PARP activating signals such as calcium signal, activating phosphorylation by certain kinases or zinc signaling may also lead to downregulation of PARP-1 activity. Of note, in the case of zinc chelators, it is not yet clear whether interference with the signaling role of zinc or simply stripping off structural zinc ions from PARP-1 results in enzyme inhibition. Some phosphorylation events are not PARP-1 activating signals but have inhibitory effects on the enzyme (for details and references see text).

Several classes of compounds with PARP inhibitory properties have been developed and characterized. Their discussion would go way beyond the scope of this review so we only refer to excellent reviews on PARP inhibitors and their therapeutic potentials [18–20]. In addition to bona fide PARP inhibitors several other molecules have been demonstrated to exhibit PARP inhibitory effects [21,22]. These include arsenite [23] (Fig. 2), tetracyclines [24], flavones, flavonoles [25], purines [26], unsaturated fatty acids [21], vitamin D3 [27], vitamin A and vitamin K [21]. However, the biological relevance of the PARP inhibitory properties of these exogenous or endogenous molecules with special regard to the relationship between their cellular concentrations and their K_i_ values remains to be determined.

A much more interesting issue is the interference between certain signaling events and PARP activation (Fig. 2). Above we discussed phosphorylation as one of the PARP-1 activating stimuli. However, phosphorylation of PARP-1 by kinases such as DNA-PK [28] or protein kinase C has been shown to inhibit the activity of the enzyme [29,30]. This finding translates to cellular consequences as it has later been demonstrated that activation of PKC by phorbol esters led to inhibition of oxidative stress-induced PARP activation and resulted in suppressed PARP-dependent cytotoxicity [31]. Calcium signal, a temporarily elevated cytosolic calcium concentration may also be required for oxidative stress-induced PARP activation [32,33]. Calcium chelators prevent oxidative stress-induced PARP-1 activation (Fig. 2), however the exact nature of the relationship between the calcium signal and PARP activity remains elusive. On the one hand PARP activity has been shown to depend on calcium [34], while this relationship is partly indirect: calcium chelation also suppressed oxidative-stress-induced DNA breakage suggesting that calcium also plays a role at a more proximal step of the oxidative stress-DNA breakage-PARP-1 activation-cell death pathway [32]. To make the situation even more complex, a late mitochondrial (?) calcium signal can also be detected in oxidatively stressed cells and this late signal responds well to PARP-1 inhibition or knockout [50].

Until recently calcium was the only ion considered to serve a second messenger role. However, similar roles have later been assigned to magnesium and zinc ions [36]. Interestingly, zinc chelation suppressed PARP activation and subsequent cell death in oxidatively stressed cells (Fig. 2) suggesting a possible link between this transition metal ion and the enzyme [37]. PARP-1 binds to DNA via its zinc fingers so it may be plausible to hypothesize that the zinc chelator withdraws the structure stabilizing zinc ion from the zinc finger motives resulting in enzyme inhibition. In light of the increasingly recognized signaling role of zinc, however, a scenario described above for calcium may not be excluded either. According to this hypothesis awaiting experimental confirmation, zinc signaling may converge on PARP-1 triggering its activation.

The role of several metabolites connected to PARP-1 including its substrate (NAD), a key metabolite required for NAD synthesis (ATP) and a product (nicotinamide) should not be disregarded as they have also been shown to affect PARP activity [38]. Discussion of these interrelationships, however, would go beyond the scope of this review.

## Cellular effects of PARP-1 activation

**Fig. 3:**
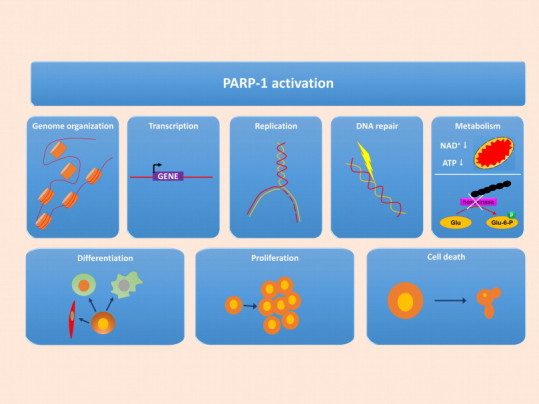
Cellular roles of PARylation. PARP-1 regulates molecular events by four mechanisms: (a) PARylation of target proteins; (b) non-covalent binding of free or protein-bound PAR polymer to target proteins; (c) protein–protein interactions between PARP-1 and partner proteins; and (d) modulation of cellular levels of NAD and ATP. These molecular events form the basis for the cellular roles of PARP-1 in the regulation of chromatin organization, transcription, replication, DNA repair and metabolism. Combinations of these cellular effects are responsible for the regulatory roles of PARP-1 in cell differentiation, proliferation and cell death (for details and references see text).

The effects of PARP-1 and PARylation are translated to cellular responses in various different ways (Fig. 3). On the one hand PARylation of PARP-1 (auto-PARylation) and other substrate proteins (hetero-PARylation) alter the physicochemical properties of the targets resulting in activation, inhibition or intracellular translocation of the modified proteins. Moreover, non-covalent binding of free or protein-bound polymer to proteins bearing one of the PAR-binding motives also affects the function of the targeted protein. Furthermore, PARP-1 can activate protein partners via protein–protein interactions and by substrate competition with other NAD-utilizing enzymes such as members of the Sir2 family of NAD-dependent deacetylases (Fig. 3).

The consequences of PARylation, PAR signaling, interaction of PARP-1 with partner proteins and substrate competition affect many cellular events ranging from genome organization [3,39], transcription [39,40], replication [41] to DNA repair [42]. These molecular pathways form the basis of such vital cellular functions such as cell proliferation [43], differentiation [44–47], metabolism and necroptotic cell death [35,48,49,51–53]. The combination of cytoprotective effects and inhibition of transcription of inflammatory mediators are considered central factors responsible for the beneficial effects of PARP inhibitors as demonstrated in various models of inflammation and tissue injury [54,55,19].

From these intertwining networks of molecular events and cellular functions here we would like to highlight one aspect bearing the highest relevance to oxidative stress: regulation of oxidative stress-induced cell death. The generally held view that in severe oxidative stress situations unrepairable DNA damage induces excessive PAR production and consequent NAD^+^ and ATP consumption has recently been challenged. Dawson's group has elegantly demonstrated [56] that PARP-1-mediated inhibition of glycolysis is not simply due to NAD consumption by PARP-1 but results from PAR binding to hexokinase the first regulated enzyme in the glycolytic pathway causing hexokinase inhibition (Fig. 3) which slows down glycolysis. The PARP-mediated cell death pathway is amenable to pharmacological intervention as proven by numerous studies reporting cytoprotection by PARP inhibitor treatment applied in various animal models of oxidative stress-related diseases [19,55].

## Conflicts of interest

Authors declare no conflict of interest.
